# An innovative strategy for deworming dogs in Mediterranean areas highly endemic for cystic echinococcosis

**DOI:** 10.1186/s13071-024-06184-x

**Published:** 2024-02-23

**Authors:** Martina Nocerino, Paola Pepe, Antonio Bosco, Elena Ciccone, Maria Paola Maurelli, Franck Boué, Gérald Umhang, Justine Pellegrini, Samia Lahmar, Yousra Said, Smaragda Sotiraki, Panagiota Ligda, AbdElkarim Laatamna, Giorgio Saralli, Orlando Paciello, Maria Chiara Alterisio, Laura Rinaldi

**Affiliations:** 1 Department of Veterinary Medicine and Animal Production, University of Naples Federico II, CREMOPAR, 80137 Naples, Italy; 2 Regional Reference Centre for Animal Health (CRESAN), Naples, Campania Region Italy; 3 ANSES, Nancy Laboratory for Rabies and Wildlife Diseases, Technopôle Agricole et Vétérinaire, BP 40009, 54220 Malzéville, France; 4 Laboratoire de Parasitologie, École Nationale de Médecine Vétérinaire, 2020 Sidi Thabet, Univ., Manouba, Tunisie; 5 Veterinary Research Institute, Hellenic Agricultural Organisation-Demeter, 57001 Thessaloniki, Greece; 6 Laboratory of Exploration and Valorization of Steppic Ecosystems, Faculty of Nature and Life Sciences, University of Djelfa, Moudjbara Road, BP 3117, Djelfa, Algeria; 7 Istituto Zooprofilattico Sperimentale del Lazio e della Toscana M. Aleandri, Rome, Italy

**Keywords:** *Echinococcus granulosus*, Control programs, Canids, Home range, GPS dataloggers, Geospatial data

## Abstract

**Background:**

Cystic echinococcosis (CE), caused by the larval stage of *Echinococcus granulosus sensu lato*, is a zoonotic parasitic disease of economic and public health importance worldwide, especially in the Mediterranean area. Canids are the main definitive hosts of the adult cestode contaminating the environment with parasite eggs released with feces. In rural and peri-urban areas, the risk of transmission to livestock as well as humans is high because of the free-roaming behavior of owned/not owned dogs. Collecting data on animal movements and behavior using GPS dataloggers could be a milestone to contain the spread of this parasitosis. Thus, this study aims to develop a comprehensive control strategy, focused on deworming a dog population in a pilot area of southern Italy (Campania region) highly endemic for CE.

**Methods:**

Accordingly, five sheep farms, tested to be positive for CE, were selected. In each sheep farm, all shepherd dogs present were treated every 2 months with praziquantel. Furthermore, 15 GPS dataloggers were applied to sheep and dogs, and their movements were tracked for 1 month; the distances that they traveled and their respective home ranges were determined using minimum convex polygon (MCP) analysis with a convex hull geometry as output.

**Results:**

The results showed that the mean daily walking distances traveled by sheep and dogs did not significantly differ. Over 90% of the point locations collected by GPS fell within 1500 mt of the farm, and the longest distances were traveled between 10:00 and 17:00. In all the sheep farms monitored, the area traversed by the animals during their daily activities showed an extension of < 250 hectares. Based on the home range of the animals, the area with the highest risk of access from canids (minimum safe convex polygon) was estimated around the centroid of each farm, and a potential scheme for the delivery of praziquantel-laced baits for the treatment of not owned dogs gravitating around the grazing area was designed.

**Conclusions:**

This study documents the usefulness of geospatial technology in supporting parasite control strategies to reduce disease transmission.

**Graphical Abstract:**

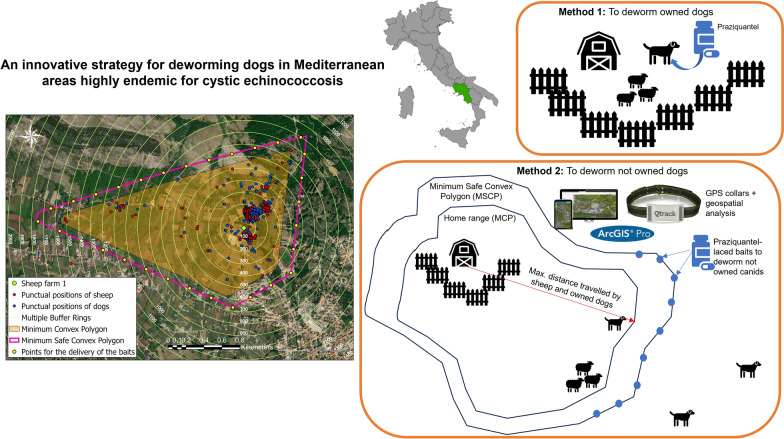

## Background

Cystic echinococcosis (CE) is a zoonotic parasitic disease caused by the larval stage of taeniid cestode *Echinococcus granulosus sensu lato*, with a considerable economic and public health significance worldwide. Recently, human CE was included by the World Health Organization (WHO) in the list of the 20 neglected tropical diseases (NTDs) and priority neglected zoonotic diseases (NZDs) for which effective control measures are needed [1, 2]. Among *E. granulosus s.l.* species, *E. granulosus sensu stricto* (*s.s.*) is the most relevant one of public health importance being responsible for 88.5% of worldwide documented human CE infections [3]. The life cycle of *E. granulosus s.s.* includes dogs (mostly shepherd dogs) as definitive hosts and small ruminants (particularly sheep) as intermediate hosts [4]. Humans act as accidental, dead-end intermediate hosts, acquiring the infection through ingestion of parasite eggs [5]. CE is highly endemic in the Mediterranean regions with high prevalence rates among communities where pastoral activities predominate [3, 6]. In southern Europe, the average annual national incidence rate of documented human CE cases ranges from one to six cases per 100,000 people, covering the years 1997–2020 [3]. Dogs, the main definitive hosts, play a pivotal role in the transmission of CE due to their free roaming in grazing areas and farms, having access to *Echinococcus*-infected offal from slaughtered livestock or by scavenging on carcasses. Eggs excreted in the environment with feces by infected canids are the source of infection for humans and other intermediate hosts [6, 7]. Compared to owned dogs, not owned ones cover a larger roaming range and thus have more access to metacestodes in carcasses or not properly destroyed infected organs, posing a higher risk of infection [8–10]. However, the contribution of stray dogs in maintaining transmission of CE has still often been neglected to date. Recently, Karshima et al. [11] conducted a meta‑analysis on the prevalence and distribution of canine *E. granulosus* infections in Africa, and the obtained results revealed the highest prevalence rates prominently in not owned dogs in North Africa. The prevalence rates reported in not owned dogs from North Africa countries range from 5.2% to 42% [12, 13]. Additionally, a prevalence of 4.2% of Taeniidae eggs, which could likely be *E. granulosus*, was reported in not owned dogs from central Italy [14]. For this reason, not owned dogs are a challenging category in the management of dog populations to control CE [11, 15], and their treatment should be much more intensive than for owned dogs [9, 10]. In this context, the “Humane Dog Population Management Guidance, 2019” [16], developed by the International Companion Animal Management Coalition (ICAM), recommended a comprehensive canine population management program, encompassing both owned and not owned dogs, based on preventive veterinary treatments such as vaccinations and parasite control to reduce zoonotic diseases, including echinococcosis. From a control perspective, canids are therefore the main targets for interventions aiming to reduce or eliminate adult worm burdens. Accordingly, praziquantel (PZQ) is an excellent cestocide for dogs, with very high and reliable efficacy against mature and immature adult stages of intestinal taeniid cestodes [17]. Over the years, control programs focusing on deworming and managing domestic dogs have been successfully implemented in several large insular areas, such as New Zealand, Iceland and Tasmania [17]. Control programs against *E. granulosus* are considered long-term measures that require an integrated approach [18]. Usually, these measures (or programs) include a combination of several strategies: (i) regulation and monitoring of slaughter activity and disposal of offal; (ii) prevention of dog access to offal from slaughtered livestock; (iii) regular deworming of dogs; (iv) public health education; (v) the introduction of EG95 recombinant vaccine for protection of lambs against *E. granulosus* infections [19, 20]. However, despite the implementation of such control initiatives in several countries and regions (i.e. Argentina, Chile, China, Italy, Morocco and Uruguay), resulting in a marked decrease in the incidence of the disease, CE remains a major public health problem worldwide [2, 18, 20–24]. Thus, new sustainable tools, especially applicable at level of definitive hosts, are needed to implement the CE control programs.

Collecting detailed data on movements and spatial behavior of hosts (definitive and intermediate) could support the planning of comprehensive control strategies. In this regard, the use of geospatial technologies and GPS dataloggers could be an optimal tool to measure animal movements with a fine spatial and temporal resolution. GPS collars have already been successfully used to evaluate the role of dog behavior in the transmission of *Echinococcus multilocularis* in the Sichuan Province of China [25]. In addition, GPS tracking data of peri-urban stray dogs have been recently used to calculate the home ranges of these animals and to investigate the spatial variation in *E. granulosus* prevalence within wild dog population in peri-urban areas of southeast Queensland, Australia [26].

Given the potential application of geospatial technologies and GPS dataloggers, the main objective of the present study was to develop and validate a comprehensive strategy for deworming both owned and not owned dogs in a pilot area of southern Italy (province of Salerno in Campania region), highly endemic for CE [16, 18], to implement control strategies against this disease.

With the primary objective in mind, the specific aims were the following: (i) implement the treatment of owned dogs on sheep farms that tested positive for CE; (ii) define the travel distances of both sheep and free-roaming owned dogs along with their spatio-temporal activity patterns; (iii) estimate the home range areas covered by sheep and dogs during their grazing activity; (iv) develop a scheme for the targeted delivery of praziquantel-laced baits to treat not owned dogs present in the grazing areas.

## Methods

### Study design

The strategies described in the present study are part of the project ECHINO-SAFE-MED “*New sustainable tools and innovative actions to control cystic ECHINOcoccosis in sheep farms in the MEDiterranean area: improvement of diagnosis and SAFEty in response to climatic changes*” (supported by PRIMA-Partnership for research and innovation in the Mediterranean area), which aims to improve surveillance and control activities for definitive (dogs) and intermediate hosts (sheep) of *E. granulosus* in four Mediterranean countries (Italy, Greece, Algeria and Tunisia) where CE is highly endemic.

**Fig. 1:**
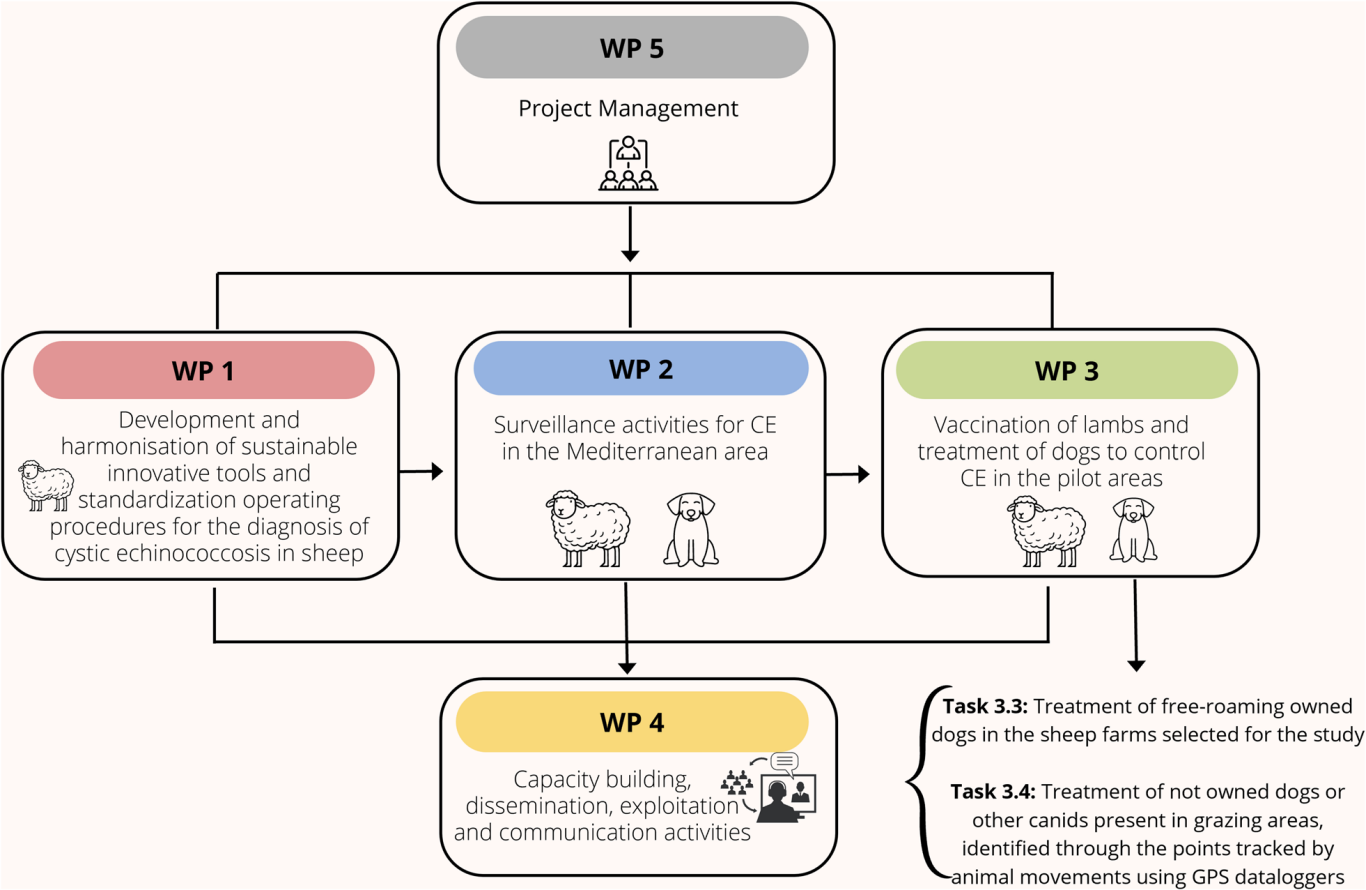
ECHINO-SAFE-MED work packages

ECHINO-SAFE-MED is divided into five inter-linked Work Packages (WPs) as described in Fig. 1. Specifically, this study was a task of WP3, which focused on evaluating new approaches to control CE, based on the treatment of definitive hosts (owned and not owned dogs) as described in detail in the following sections. In addition to the conventional control activities, an innovative strategy based on praziquantel-laced baits was designed for use in the treatment of not owned dogs or other canids in grazing areas identified by tracking animal movements through GPS dataloggers.

Activities have been ongoing since August 2021 in a pilot area of southern Italy highly endemic for CE [16, 18] to evaluate whether it is feasible to extend the use of these technologies to other countries of Mediterranean area.

### Study area and recruitment of sheep farms

The study was conducted in a peri-urban area of the Salerno Province (Campania region, southern Italy), where owned and not owned free-roaming dogs are common. To select the sheep farms to be included in the study, in-depth interviews were conducted with farmers in the pilot area to gather information on farm management practices. Special attention was paid to selecting representative farms (e.g. similar farm size, breeding systems, number of shepherd dogs). To use these data while protecting the privacy of people (i.e. farmers), farm(ers) names, ID and addresses were transformed into anonymous data using progressive numbers. Each farmer received information about the research and how their data were used. A letter of consent was filled by each farmer involved into the project.

Based on the questionnaire survey, a total of 40 sheep farms with approximately 200 animals each were selected and underwent surveillance activities, using both ultrasound (US) [27] and post-mortem examinations. The results obtained were collected and analyzed, and 10 sheep farms, with a mean intra-flock prevalence of CE > 30%, according to the US analysis, were selected for the study. Specifically, five sheep farms were selected to start the treatments of dogs (owned and not owned) whereas the other five sheep farms were selected to be used as control farms without any treatment activities for dogs.

**Fig. 2:**
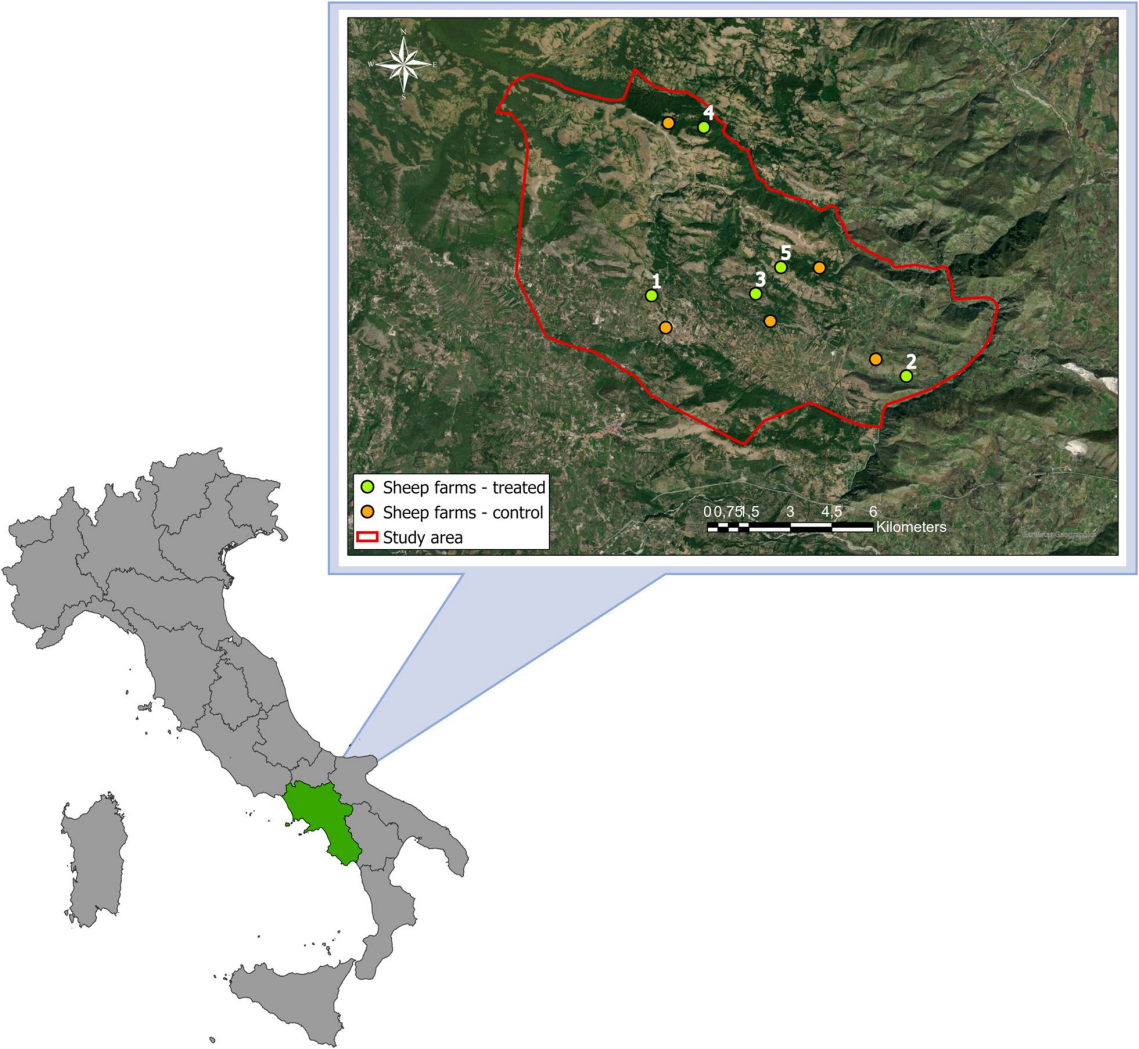
Localization of the five sheep farms (green points) in which the treatments of dogs (owned and not owned) were carried out and the five control farms (without treatment of dogs) (orange points), selected for the pilot study in the Salerno province, Italy

The location of the sheep farms included in the study was then geo-referenced using a Geographical Information System (GIS) (ArcGIS Pro 2.7 software, Environmental Systems Research Institute, Inc., Redlands, CA, USA) (Fig. 2).

### Treatment and diagnosis of dogs in sheep farms

The shepherd dogs in each sheep farm included in the study were treated and underwent post-treatment parasitological examination. Treatments were performed orally with chewable tablets containing a combination of praziquantel (5 mg/kg) and milbemycin oxime (0.5 mg/kg) (Milbemax®-chewable tablets, Elanco Italia S.p.A). The intervention interval was set at 2 months for the entire duration of the project. To facilitate the treatment of the dogs and collection of fecal samples, specifically designed and purpose-built modular mobile cages (EchinoCage) were used, as previously described [18]. After 48 h, all fecal samples were collected individually using the Fill-FLOTAC [28] while confinement areas of the dogs were disinfected with 5% sodium hypochlorite to avoid environmental contamination by Taeniidae eggs [18].

Fecal samples were stored at − 80 °C for 3 days prior to copromicroscopic analysis to inactivate *E. granulosus* eggs [29].

The Mini-FLOTAC technique [18], with zinc sulfate (specific gravity = 1.35) as flotation solution [30], was used to detect and count Taeniidae eggs (analytical sensitivity = 5 eggs per gram of feces, EPG). All fecal samples testing positive for Taeniidae eggs underwent molecular analysis to detect *E. granulosus* s.s.

The molecular diagnostic was realized from 300 mg of fecal sample mixed with 1 ml of CTAB buffer in a Lysing Matrix E tube (MP biomedicals) before heating 5 min at 95 °C. After cooling, the tube was submitted to three grinding cycles in the FastPrep (MP biomedicals) with one cycle composed of 1 min at 5 m/s followed by 1 min on ice. The DNA extraction was then continued as recommended using the Maxwell 48 with the Maxwell RSC PureFood Pathogen Kit protocol (Promega). A real-time PCR multiplex, combining the detection of *E. granulosus* *s.s.* with primers and probe as described by Maksimov et al. [31], and an internal control, which is a plasmid artificial construct allowing the amplification with the same primers as for *E. granulosus s.s.* but requiring a specific probe, was performed. The reaction was performed in duplicate with Maxima Probe qPCR Master Mix (ThermoScientific) in a final volume of 25 μl, including 5 μl of DNA from the fecal sample, and run on a QuantStudio5 thermocycler (Applied Biosystems). The final concentrations of 1.3 μM for primers and 0.2 μM and 0.1 µM were used for the probes of *E. granulosus* *s.s.* and the internal control, respectively. One hundred copies of the internal control were added in each tube. The multiplex qPCR program used was 10 min at 95 °C and then 45 cycles of 15 s at 95 °C and 60 s at 60 °C.

Additionally, the presence of *Taenia* species was detected using PCR primers Cest4–Cest5 targeting the 12S mitochondrial gene as described by Trachsel et al. [32]. A private company (Eurofins Genomics, Germany) sequenced the amplicons obtained through conventional PCR, while the Geneious prime software was used to align nucleotide sequences.

### GPS tracking data and identification of points for the delivery of praziquantel-laced baits

**Fig. 3:**
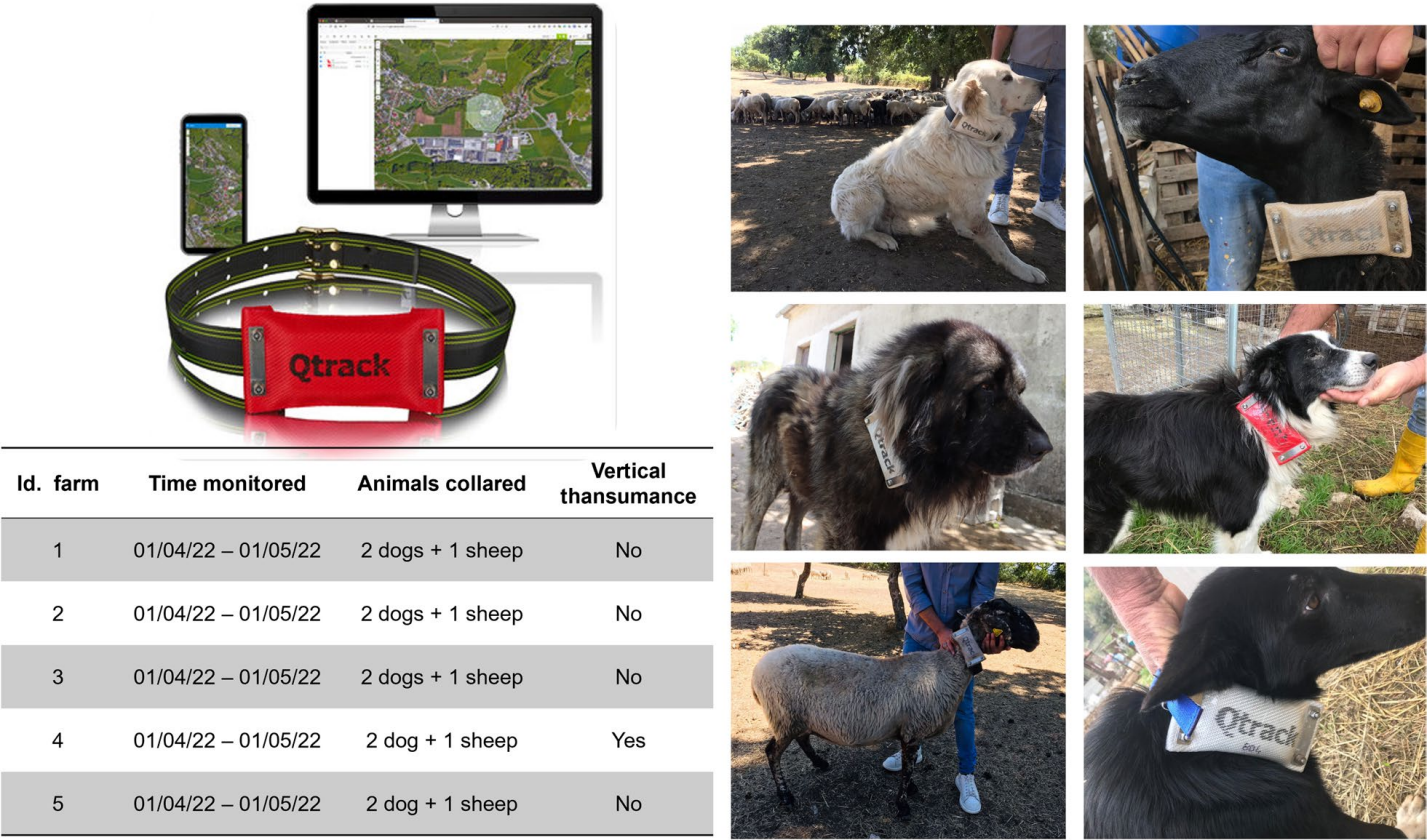
GPS devices used for tracking sheep and dog movements

On each sheep farm selected for the treatment, three GPS datalogger collars (Qtrack GPS, 4G LTE Iot Network Technology, Austria) were attached to the sheep acting as “flock leader” and to two shepherd dogs to monitor animal movements. The movements of sheep and dogs were monitored at the same time for 1 month (April) on all the farms (Fig. 3). GPS devices were selected based on their suitability for field studies, which included ease of programming, light weight (140 g), small size (87 × 51 × 30 mm), battery capacity of 2400 mAh, long battery life (> 2 months) and water resistance. Since the GPS devices used the Global System for Mobile Communication (GSM) to transmit the acquired locations to the server, the mobile network coverage over the entire pilot area was verified before starting the tests.

The GPS devices were programmed to record the geographic coordinates of the animals every hour. Before starting GPS tracking, signal reception and network coverage were checked in two different typical micro-habitats of the study area (agro-forestry and natural grassland) by installing four GPS collars on medium-sized shepherd dogs that were allowed to roam freely for 5 h in an area of 3 hectares (ha). The point locations recorded at 1-h intervals for the preliminary test showed an accuracy of up to 3 mt.

Only on one sheep farm (ID 4), where animals were performing vertical transhumance, two pens at different altitudes (4 M: high altitude pen, 4 V: low valley pen) were monitored.

Positions recorded by the GPS dataloggers were downloaded, grouped per animal species (sheep and dogs) and processed using Microsoft Excel (Microsoft Corp., Redmond, WA, USA) for further statistical analysis (see below). Only data points which included all the information (i.e. date, time, latitude and longitude) were selected.

A GIS platform was developed using ArcGIS Pro 2.7 software (Environmental Systems Research Institute, Inc., Redlands, CA, USA) to project the point locations onto the study area.

For each farm, the daily walking distances (mt) traveled by sheep and dogs were calculated by summing the Euclidean distance between consecutive GPS point locations collected in a day. Then, these values were averaged over 1 month to calculate the mean daily walking distance (mt/day).

A multiple ring buffer analysis was implemented to estimate the size and spatial distribution of the positions of collared animals. Ten concentric buffers were created around the centroid of each farm, spaced 100 mt apart, and the number of sheep and dog point locations which crossed each ring buffer was counted. The area traversed by sheep and dogs during their daily activities (home range) was determined using minimum convex polygon (MCP) analysis with a convex hull geometry as output. The estimation of the home range area was performed considering 100% MCP method [33] to identify the maximum extension of the grazing area. To protect the grazing area from the intrusion of potentially infected animals, a minimum safe convex polygon (MSCP) was constructed around the centroid of each farm by enlarging the MCP within the circular area with a radius of 5 km, which is assumed to be the home range for free-roaming canids [8]. On the perimeter of these surfaces, points for the delivery of the anthelmintic treatment were fixed. At each point, highly attractive baits laced with 125 mg praziquantel + 12.5 mg milbemicine [34] were manually disseminated at 2-month intervals, maintaining an average bait density of 20–25 baits/km^2^ [35]. Every 2 months, praziquantel-laced bait application sites were advanced 50 mt on the MSCP to distribute the drugs evenly along the perimeter of the grazing area.

### Statistical analysis

A multi-distance spatial cluster statistical analysis, based on Ripley's K-function, was implemented to investigate how the spatial dependence (clustering/dispersion) of sheep and dog point locations changed at different distances from the farm (0–3000 mt; distance increment: 100 mt) [36]. For each farm, t-test statistical analysis was conducted to establish whether there was a significant difference between the mean daily walking distances traveled by sheep and dogs. In addition, the distances of the point locations from the centroids of the farms resulting from the multiple ring buffer analysis were related to the temporal data to identify daily peak activity patterns of the animals; Pearson statistical analysis was conducted to test the significance of these spatio-temporal correlations. All statistical analyses were performed with IBM SPSS V.26 statistical software package. *P* < 0.05 was considered statistically significant.

## Results

### Treatment and diagnosis of dogs in sheep farms

A total of 27 shepherd dogs were recorded on the five treated sheep farms, with an average of 5.4 dogs per farm (minimum 3–maximum 6). All dogs had received anthelmintic treatment with praziquantel at 2-month intervals. Taeniidae eggs were found in two fecal samples only in one sheep farm directly after the first treatment (T0) with praziquantel (mean EPG = 842), which did not correspond to *E. granulosus* *s.s.* but one to *Taenia hydatigena* and the other to *T. pisiformis*. Since T1 treatment, none of the fecal samples, analyzed every 2 months, tested positive for Taeniidae eggs.

### GPS tracking data and identification of points for the delivery of praziquantel-laced baits

Due to unstable network coverage, a total of 6973 point locations were recorded. Data were extracted from 13 of 15 GPS installed devices, since two collars (GPS installed on two shepherd dogs belonging to farms 1 and 5) became unusable during the study. The number of point locations acquired ranged from 285 to 642 for sheep and from 242 to 1204 for dogs.

In each sheep farm, > 90% of sheep and dog point locations were within 1500 mt from the centroid of the farm but the core areas of roaming space used for sheep and dogs were found to be within 500 mt (70%) (Table 1). These results were confirmed by multi-distance spatial cluster analysis, which reported the presence of statistically significant clusters of sheep and dog punctual positions over a range of 0–500 mt distance from the centroid of the farm, with a confidence envelope value equal to 99%.

**Table 1:** Point locations of sheep and shepherd dogs classified per categories of multiple buffer rings at fixed distance of 500 mt

Farm id.	Animal species	Distance from the centroid of the farm (mt)						
		*d* ≤ 500	*d* ≤ 1000	*d* ≤ 1500	*d* ≤ 2000	*d* ≤ 2500	*d* ≤ 3000	Total
1	Sheep Dog	598 956	21 27	19 21	4 10	0 3	0 0	642 1017
2	Sheep Dog	362 1114	10 43	35 47	0 0	0 0	0 0	405 1204
3	Sheep Dog	527 877	63 94	14 29	0 0	0 0	0 0	604 1000
4 M*	Sheep Dog	285 273	11 19	9 6	2 1	0 6	0 0	307 305
4 V*	Sheep Dog	248 193	32 40	5 7	0 2	0 0	0 0	285 242
5	Sheep Dog	427 480	31 24	0 0	0 0	0 0	0 0	458 504

^*^(4 M: high altitude pen, 4 V: low valley pen)

**Fig. 4:**
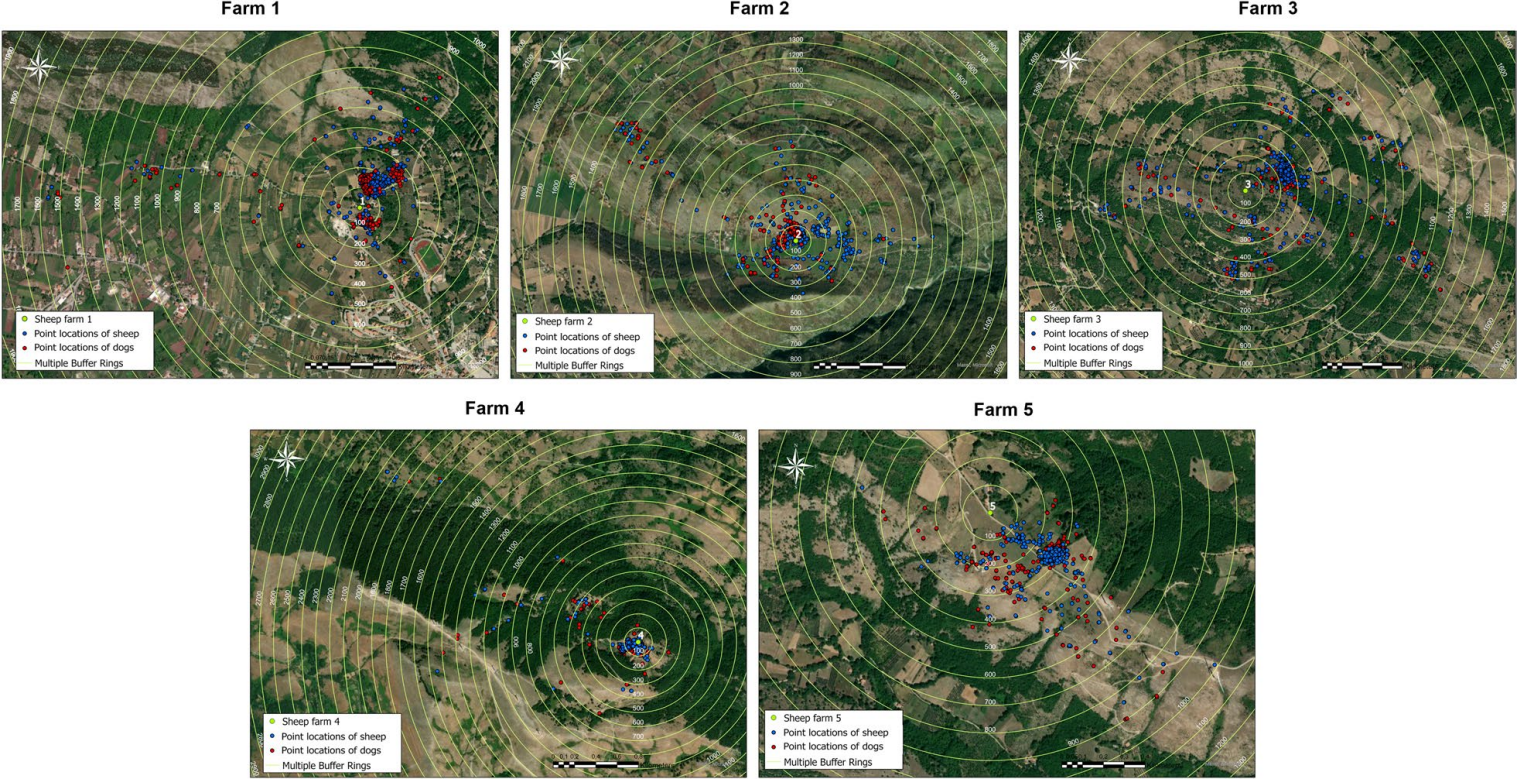
The spatial distribution of the point locations of sheep (red points) and dogs (blue points) logged by GPS dataloggers in the five studied farms. The multiple concentric buffer rings (100 mt) provide the measurement of the animal distance from the centroid of the farms

Sparsely frequented zones were found in all grazing areas, alternating with zones of high density of point locations (Fig. 4).

In the five sheep farms monitored, the extent of the home range areas of sheep and dogs was ≤ 250 ha; the farthest point locations recorded were 2004 mt and 2500 mt for sheep and dogs, respectively (Table 2).

**Table 2:** Spatial parameters of sheep and dog populations for each examined farm

	Farm id.					
	1	2	3	4 M	4 V	5
Home range (hectares)	132	129	150	178	250	51
Maximum distance traveled by sheep (mt)	2004	1412	1343	2001	1305	1000
Maximum distance traveled by dogs (mt)	2510	1436	1325	2210	1720	1100
Mean daily walking distance traveled by sheep (mt)	1302	1722	1895	1929	1938	1028
Mean daily walking distance traveled by dogs (mt)	1585	1897	1715	2430	1931	1030

No significant differences in the mean daily walking distance traveled by sheep and dogs were found across the examined farms. This distance ranged from 1028 to 1938 mt for sheep and from 1030 to 2430 mt for dogs (*P* > 0.05).

The t-test analysis conducted to test the difference between the mean daily distances traveled by sheep and dogs showed no significance (*P* = 0.7).

### Spatial-temporal distributions of sheep and dog point locations

The analysis of the spatio-temporal profiles of sheep and dog point locations revealed that they were most active during daytime hours (06:00–18:00). Since in each sheep farm the point locations of the two collared dogs were not significantly different (*P* > 0.05), the data points of only one dog were compared to sheep data points for the construction of the spatio-temporal profiles.

**Fig. 5:**
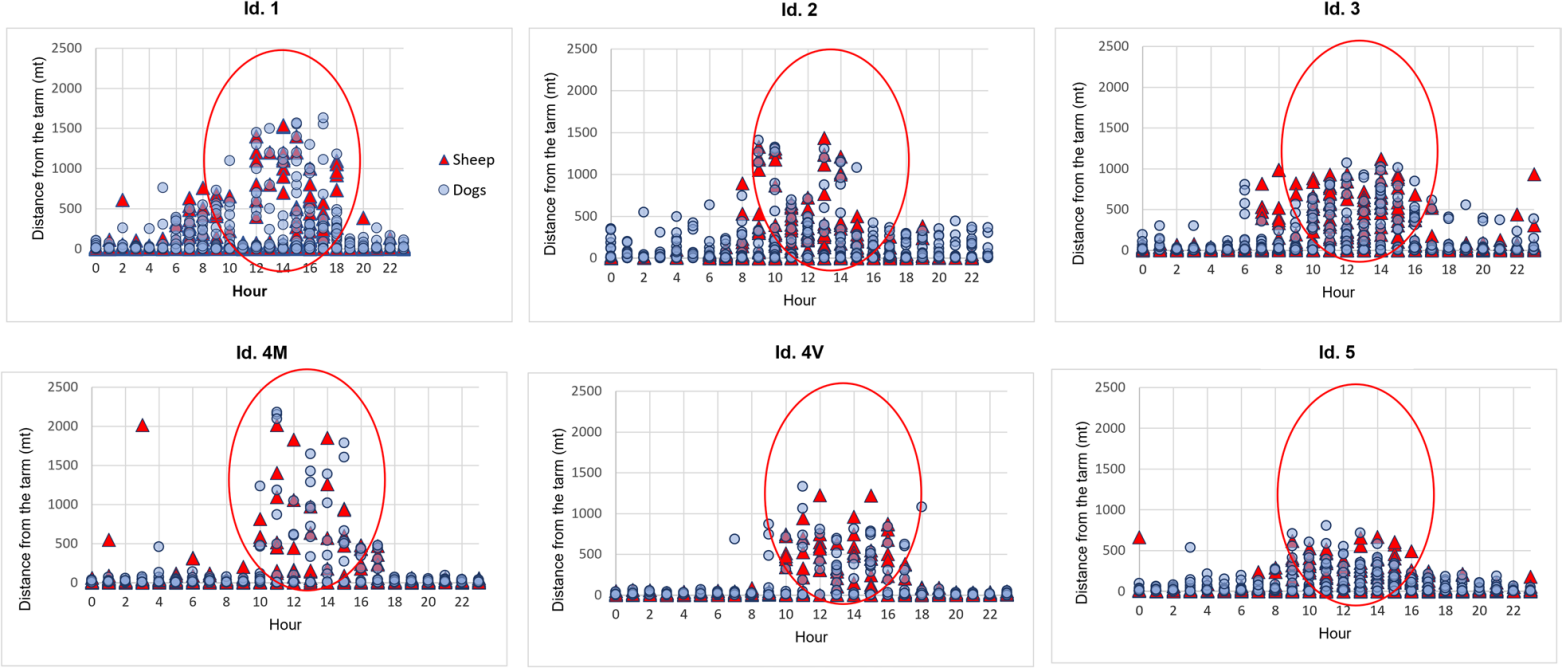
Example of spatio-temporal activity patterns produced by the positions of sheep (red triangles) and free-roaming owned dogs (blue circles). The point locations collected by GPS devices between 06:00 and 17:00 were classified according to the time of day (horizontal axis) and the distance from the centroid of the farm (vertical axis)

All the examined farms were characterized by the same management of the grazing activity: out of the stable and start of the activity between 06:00 and 07:00 in the morning, greatest distances from the centroid of the farms generally covered during the 10:00–17:00 time slot and return to the farm between 17:30 and 18:00 (Fig. 5). Pearson’s test conducted on the spatio-temporal correlations of sheep and dog movement data showed low values in each farm analyzed (Pearson’s coefficient: 0.25–0.43; *P* = 0.05). No significant differences in peak temporal activity periods between sheep and dog movements emerged.

**Fig. 6:**
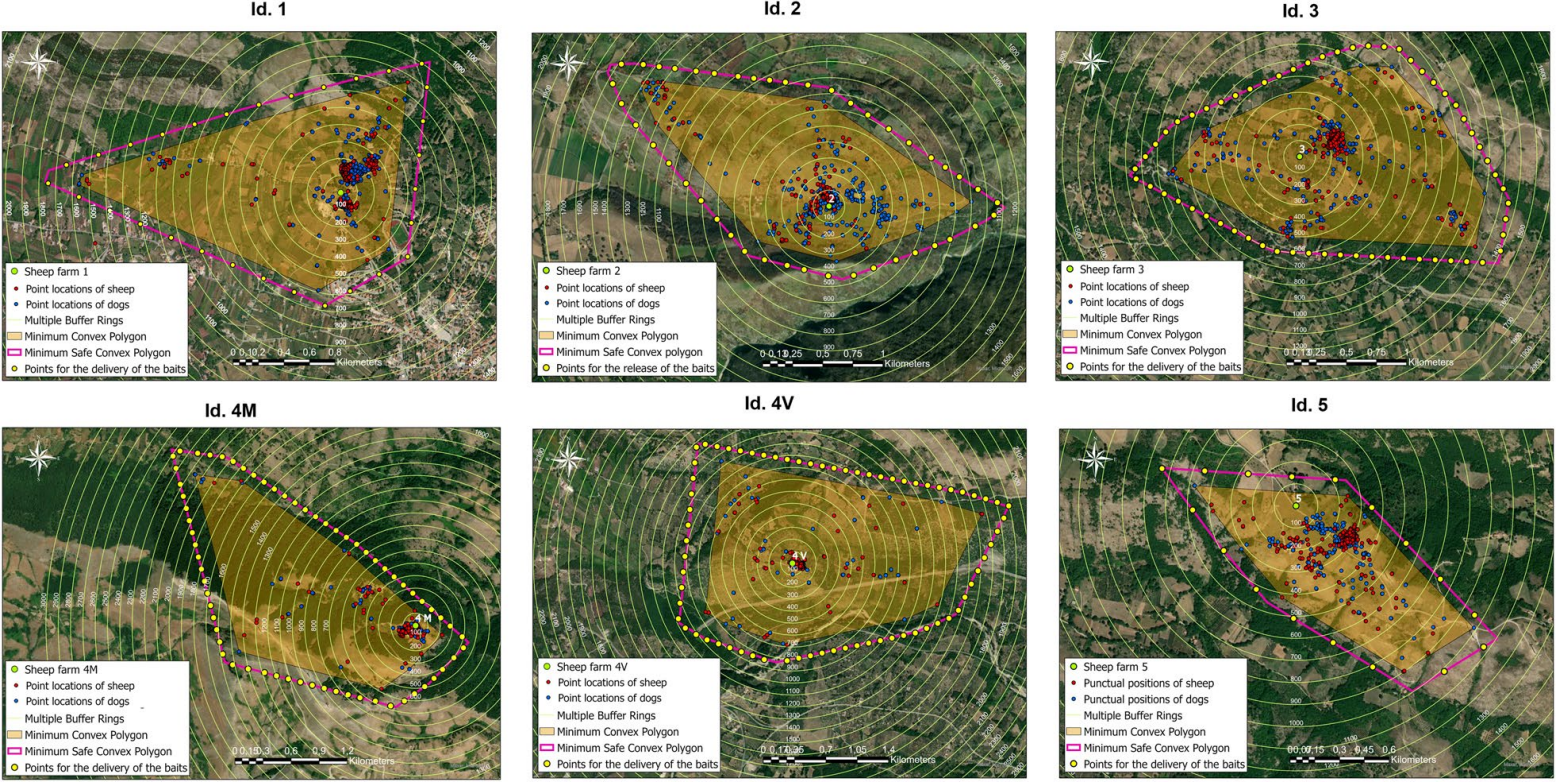
Home ranges of sheep and dogs estimated using minimum convex polygons (orange area). The points for the delivery of the medicated baits (yellow points) for the treatment of stray canids were fixed on the boundaries of the minimum safe convex polygon (pink polygons)

Figure 6 shows the home range (MCP) calculated for each sheep farm, considering sheep and dog point locations. The area with the highest risk of access from stray canids was estimated for each farm (MSCP), and a potential scheme for the delivery of praziquantel-laced baits was designed. The number of points for the delivery of the baits ranged from a minimum of 40 to a maximum of 70, depending on the extension of the MCP, and were positioned at a fixed distance of 100–200 mt.

## Discussion

The present study has illustrated a comprehensive strategy for deworming owned and not owned dogs, developed and validated in a Mediterranean area highly endemic for CE. This strategy could be included in the control programs for CE with the final aim to significantly reduce the transmission of this parasitosis. Since the mid-nineteenth century, the public health importance of CE has been recognized, and considerable efforts have been made to reduce the disease [19, 20]. However, despite the implementation of such control initiatives in several countries, CE remains a problem in the Mediterranean areas with a high rate of infection. One of the factors statistically associated with the perpetuation of CE in endemic areas is the presence of stray dogs and their free access to carcasses of intermediate hosts [37]. This was supported in this study, where *E. granulosus* was not detected in any shepherd dogs belonging to the five sheep farms with animals testing positive for CE, confirming the involvement of stray canids in the transmission of CE.

While the regular treatment of owned dogs using an effective cestocidal anthelmintic such as praziquantel is possible [38], as also demonstrated in this study, the treatment of free-roaming populations of unowned dogs is extremely difficult [15]. Thus, new sustainable tools for the treatment of stray canids are needed to implement CE control programs. In this regard, the control approach described here is based on a high spatial resolution analysis of the sheep and dog movements using innovative GPS dataloggers combined with traditional and innovative treatment strategies for owned and stray dogs, respectively.

In recent years, thanks to breakthroughs in GPS-based technologies, including improved precision, lower costs and greater battery efficiency, there has been growing use of these technologies to precisely collect large data sets on the positions of individuals in the landscape [39]. In particular, several studies have demonstrated the potential for using GPS tracking to study animal behaviors and their interaction with the environment, and how these affect parasite transmission [40]. In this regard, a preliminary analysis was done to define the movements of sheep and dogs, belonging to CE farms, and their spatio-temporal activity patterns. The results obtained showed that the distances traveled by collared sheep and shepherd dogs were comparable in terms of mean daily walking distance with a maximum value of 2.4 km. A much longer daily walking distance (13 km) was reported when, for example, dogs had to scavenge for food or follow owners in their daily commitments [41], as shown by Mutwiri et al. [42] in a study conducted with 10 free-roaming owned dogs collared with a GPS tracker in the pastoral communities of Kenya. Concerning the temporal activity of sheep and shepherd dogs, few data have been published yet. According to Sparkes et al. [43], in this study, sheep and dogs were active from 06:00–20:00 with a wide peak activity period in the middle of the day (10:00–17:00), in contrast to other studies [33, 44, 45] in which animals exhibited two peak activity periods during the day (07:00–10:00, 16:00–19:00), probably because of different management systems on the farms. However, in this study, the variability of animal space use across seasons was not estimated, since the movements of sheep and dogs were monitored only for 1 month. Further studies need to be undertaken to investigate the interplay between animal behavior and *Echinococcus* to effectively define strategies to mitigate parasite exposure.

The GPS point locations acquired were also used to construct the home ranges of the animals within the grazing areas. The home range was defined as bounded regions representing the areas used by the animals for some purpose with different rates of usage by individuals [39]. For almost all the farms (farms 1, 2, 3, 4 M) the home range areas of the collared animals did not differ significantly (mean = 168.4 ha, range = 51–250), except for farm 5 which showed a reduced extension of the home range (50 ha) probably due to fewer potential attractions (e.g. houses, farms) surrounding the grazing area. The mean home range resulting from this study was in accordance with the data published by Sparkes et al. [46] (mean = 290.1, range = 0.8–1776.2 ha). Moreover, collared shepherd dogs spent most of their time within a few meters of the farm centroids (10–500 mt), as reported for community dogs in western China (10–250 mt) [25] and roaming dogs of rural communities in southern Kyrgyzstan (11–931 mt) [47] as a confirmation of the fact that a fixed home base and food provided by owners reduces the movements of the dogs [46].

The estimation of sheep and dog home ranges proved very useful to design a scheme for the delivery of praziquantel-laced baits for the treatment of not owned canids gravitating around the farms. In a previous study conducted in the Autonomous Prefecture in Sichuan by Yu et al. [48], to deworm stray dogs and wild canines using praziquantel-laced baits, the area was divided in a mesh of 20 × 100 mt units, each containing a fixed point for bait delivery. In contrast, in this study, MSCPs were designed for the delivery of anthelminthic baits on the border zone of the study area; these boundaries follow the trends of flocks and were positioned sufficiently far from the home range areas (MCP) to avoid over-dosing of the shepherd dogs already treated. In general, it is suggested that in a bait-based strategy, spacing of baits should be within the radius of attraction to baits, i.e. the average distance between an animal’s home range centroid and bait sites that they visit [49].

The distribution of praziquantel-laced baits along the area adjacent to that of pasture could represent a good strategy to limit the intrusion of infected animals in the grazing area, considering that the targets of the treatment were mainly not owned canids gravitating around the sheep farms. As the life cycle of *E. granulosus* also includes wild species as definitive and intermediate hosts, application of praziquantel-laced baits against wild and stray canids could lead to a significant reduction in CE prevalence. In several European contexts, including the study area, it is well known that the life cycles of *E. granulosus* and other important cestodes often overlap with wildlife, resulting in somewhat semi-domestic life cycles [50]. It has also been shown that wild boar carcasses may contain many metacestode specimens, thus acting as an infection reservoir for a potentially large number of wild canids [51]. Another aspect that should be considered is the role of hunting dogs in the transmission of CE during hunting activities. Many wild boars are hunted in the study area, and a high prevalence of metacestodosis has been reported in wild boar populations [52, 53], which could confirm the role of hunting dogs in maintaining a sylvatic cycle of CE and the importance of targeted treatments.

In several European countries, the distribution of praziquantel-laced baits for removing the adult form of *E. multilocularis* from wild canids, in endemic urban [54, 55] and rural [56] areas, has already yielded encouraging results [57], showing a considerably lower contamination of foxes with *Echinococcus* eggs [57].

Although it has already been demonstrated that praziquantel baiting campaigns against *E. granulosus* with a frequency > 1 month are less effective because of the parasite’s prepatency period [56, 58], in this study a 2-monthly frequency of treatment was proposed to test the efficacy of a control measure also applicable in less-developed countries, where the lack of economic and logistical resources makes the monthly treatment not sustainable.

The dissemination of the drug in the environment could be a problem for other animal species accidentally ingesting the baits and for the ecosystem itself. However, PZQ is a very safe drug, and overdosing or inadvertent treatment of nontarget species usually does not cause problems [15, 59].

One of the main factors influencing the effectiveness of a baiting campaign is the use of attractive baits to ensure bait uptake by canids. Therefore, highly palatable praziquantel-laced baits, with double-layer coverage, were used in this study. Specifically, Ciccone et al. [34] showed that these types of bait are highly resistant over time (up to 10 days) and to different climatic conditions, preserving their palatability for dogs. Praziquantel-laced baits were at first manually released. The next step will be the use of unmanned aerial vehicles (UAVs), designed for the ECHINO-SAFE-MED project [60], for the delivery of baits to deworm stray canids as already tested by Yu et al. [48] in areas highly endemic for alveolar echinococcosis (AE) in China. This methodology has the potential to help cut cost and labor needs in areas highly endemic for echinococcosis and makes it possible to access hard to reach grazing areas. A limitation of this study is that the prevalence of *E. granulosus* was not determined before starting the treatment so there was no initial value with which to compare subsequent measurements.

An additional analysis to evaluate the extent of soil contaminated by *E. granulosus* eggs could be useful to obtain information about a potential reduction of exposure of sheep to infection by eggs released with feces in the environment. Recently, Da Silva et al. [61] showed that analyses of feces and soil complement each other to describe the contamination of vegetable gardens by *E. multilocularis*, with the advantage of being able to design soil sampling in advance, which reflects long-term contamination when feces are only a proxy assessing an instant indication of environmental contamination.

## Conclusions

The present study confirms the usefulness of geospatial technology in supporting parasite control strategies and demonstrates that the collection of detailed data regarding the movements and the behavior of animals might be a useful method to interrupt the *Echinococcus* life cycle and to reduce the spread of the disease. The outcome of this approach will be evaluated for 4–5 years following the initiation of the activities through the assessment of (cystic) echinococcosis infection levels in sheep and dogs belonging to the selected farms (treated and controls).

The newly developed strategy could be part of an integrated control program against CE, combining anthelmintic dog treatment with livestock vaccination and public health information.

## Data Availability

Not applicable.
